# Acne Vulgaris, Atopic Dermatitis and Rosacea: The Role of the Skin Microbiota—A Review

**DOI:** 10.3390/biomedicines10102523

**Published:** 2022-10-09

**Authors:** Giorgia Condrò, Marta Guerini, Michela Castello, Paola Perugini

**Affiliations:** 1 Department of Drug Sciences, University of Pavia, Via Taramelli 12, 27100 Pavia, Italy; 2 Centro Medico Lombardo, Piazza Dante 1H, Via Melchiorre Gioia, 27100 Pavia, Italy; 3 Centro Medico Lombardo, Via Melchiorre Gioia 37, 20124 Milano, Italy; 4 Etichub, Academic Spin-Off, University of Pavia, Via Taramelli 12, 27100 Pavia, Italy

**Keywords:** skin bioengineering, skin disease, dermatitis, acne vulgaris, rosacea

## Abstract

The skin harbors a huge number of different microorganisms such as bacteria, fungi and viruses, and it acts as a protective shield to prevent the invasion of pathogens and to maintain the health of the commensal microbiota. Several studies, in fact, have shown the importance of the skin microbiota for healthy skin. However, this balance can be altered by intrinsic and extrinsic factors, leading to the development of skin disease, such as acne vulgaris (AV), atopic dermatitis (AD) and rosacea(RS). Although these diseases are widespread and affect both adolescents and adults, the scientific correlation between these disorders and the skin microbiota and physiological parameters (TEWL, hydration and lipid composition) is still unclear. This review aims to investigate the current literature regarding the correlation between the skin microbiota and its imbalance underlying microbiological aspects, how the skin microbiota changes over the course of the disease and the current possible treatments. The following reported studies show a general imbalance of the bacterial flora. For this reason, more in-depth studies are necessary to explore the different subspecies and strains involved in all three diseases.

## 1. Introduction

**Figure 1:**
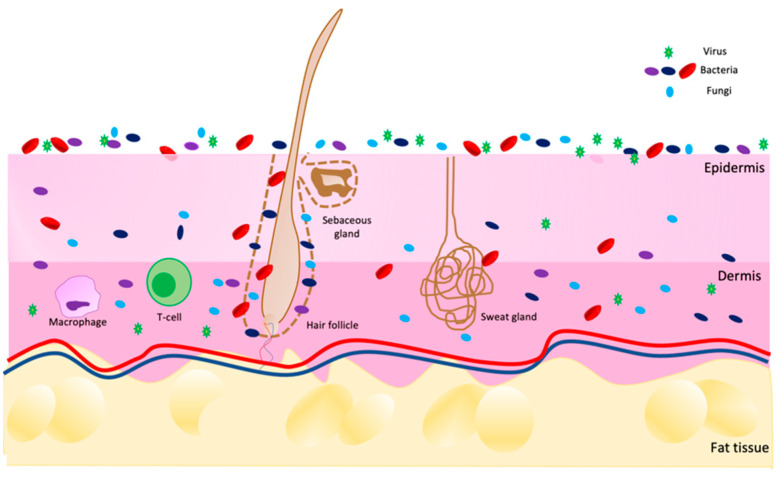
Scheme of skin microbiota distribution on skin in a healthy condition. Image built on Power Point software.

The skin is an extensive and dynamic system in which different living microorganisms (bacteria, fungi, viruses, and mites) populate not only the surface but also the deeper layers of the epidermis and dermis, as shown in Figure 1. The entirety of the population composes the microbiota [1,2,3]. This term refers to all commensal microorganisms, which are usually harmless [4], present in and on our bodies, such as in the intestine, nose, mucous membranes, scalp and skin.

Their function is to defend our body from pathogens and to maintain good health over time [5,6]. To keep a good balance, it is necessary that different microbial communities participate in a symbiotic relationship with the host tissue, conferring it some benefits [7]. In fact, the microbiota includes two types of microorganisms: resident and transient. The first group represents the core of the skin environment, and they help to maintain healthy conditions. In fact, they are neither aggressive nor pathogenic, but they provide some benefits to the host, also preserving the skin barrier. The latter, as the name suggests, do not stabilize permanently, but remain on the skin for only a short period. Under normal conditions, they are not pathogenic.

However, exogenous and endogenous factors can perturb the bacterial flora, leading to changes in the relative abundance of normal commensals, and these could become opportunistic pathogens under the right circumstances [8], contributing to the symptoms [1,2] of diseases such as acne vulgaris (AV), atopic dermatitis (AD) and rosacea (RS) [9,10,11]. 

The distribution of the skin microbiota is not homogeneous, but it differs according to body areas and to skin characteristics. At the macroscopic level, we can subdivide moist, dry, and sebaceous environments. In the sebaceous areas, we find lipophilic bacteria such as *Cutibacterium,* which is able to grow well in anaerobic, lipid-rich regions (for example, the forehead and nose crease). In dry areas, on the other hand, *Micrococcus, Streptococcus*, and *Corynebacterium* prevail, such as in the upper buttock area and forearm. 

At last, *Staphylococcus* and *Corynebacteria* species prefer moist areas, e.g., the armpit, inguinal fold and inner elbow [8,12]. 

If we consider smaller body regions, at the microscopic level (eccrine and apocrine glands, sebaceous glands, and hair follicles), more microbial heterogeneity is present. *Cutibacterium* species predominate, for example, in sebaceous follicles. 

In addition to microbial heterogeneity, bacterial flora also varies from individual to individual by birth type. In the beginning, the fetus in utero is sterile, so the skin microbiota begins to develop at the time of birth. If the childbirth method is cesarean, then the newborn will have a microbial population similar to the mother, and species of *Staphylococcus, Corynebacterium,* and *Cutibacterium* will prevail. If, on the other hand, the childbirth is natural, then the baby will have a vaginal microbial set, such as *Lactobacillus, Prevotella,* and *Sneathia* [13]. 

Then, the enrichment of the microbiota continues with the lactation phase, during which the mother’s microorganisms try to reach all areas of the body, including hair follicles and then the scalp, in order to establish a healthy relationship with the skin cells of the host. Adulthood is then the final stage in which everyone will have their own bacterial flora that will not be static, but will change over time. These temporal changes are some other factors affecting interindividual and intraindividual variability. In a study conducted over the course of 4–6 months, it was observed that microbial dynamism was greater in dry environments than in mixed environments.

In addition, other factors that increase the variability of microbial flora are lifestyle, work, race and age. During puberty, young people have greater sebum production, which consequently correlates with a greater presence of lipophilic bacteria such as *Cutibacterium.*

Another aspect to consider is the environmental impact, including climate, temperature, and UV exposure. Indeed, UVA and UVB radiation are bactericidal [8,12].

Under healthy conditions, the most prevalent bacteria phyla are *Actinobacteria, Bacteroidetes, Firmicutes* and *Proteobacteria*; in particular, the three most common genera are *Corynecateria, Cutibacteria* and *Staphylococci* [13,14,15,16]. 

Among the commensal bacteria, the most representative are *Cutibacterium acnes*, *Staphylococcus epidermidis* and *Corynebacterium jeikeium*.

*Cutibacterium acnes*, a Gram-positive anaerobe bacterium, resides in the sebaceous areas of the body, such as the face, neck, hair follicles and sebaceous glands. Other species, such as *C. avidum* and *C. granulosum*, are also found, but in smaller quantities. *Cutibacterium acnes* is considered one of the main commensal bacteria of the skin; in fact, it metabolizes fatty acids with antimicrobial properties and contributes to maintaining the acidic pH of the skin. It also produces bactericides, preventing the growth of yeasts, molds and some Gram-negative pathogens. However, under certain conditions, some subspecies of *C. acnes* are implicated in acne (which will be further explained later in this article) [15,16].

*Staphylococcus epidermidis*, which is Gram-positive, belongs to the *Firmicutes* phylum. It is commonly found on the skin and is considered beneficial: it is able to secrete antimicrobial peptides (AMPs), such as epidermin and epilancin K7, which prevent the colonization of skin pathogens, including Group A Streptococci (*S. pyogens*) and *S. aureus.*

Corynebacteria, Gram-positive bacteria (phylum Actinobacteria), colonize moist or sebaceous sites and use lipids or vitamins from sweat to survive.

Members of this family include *C. jeikeium*; this is a commensal bacterium that, in much the same way as *S. epidermidis*, produces bacteriocin-like antimicrobial compounds, preventing the colonization of other potentially harmful species. In addition, *C. jeikeium* also produces superoxide dismutase, an enzyme that protects bacteria from superoxide radicals and simultaneously may prevent oxidative damage at the tissue level [17,18]. 

The skin microbiota does not include only bacteria but also fungi. Indeed, different studies have suggested that the most present fungi on the skin belong to the *Malassezia* species, such as *M. globosa*, M. *restricta* and M. *sympodialis*. The distribution of these microbes is body-dependent; for example, *M. globosa* lives on the back and occiput, whereas *M. restricta* is instead on the external auditory canal and retroauricular fold. *Aspergillus, Rhodotorula, Cryptococcus* and *Epicoccum* are present in other skin areas, such as foot sites [17]. 

The field of viruses, on the other hand, is not completely known. This may be due to the difficulty of sampling and sequencing them, for example, due to their size [18]. 

This review aims to investigate the bacterial population usually present on our skin and the microbial changes in pathogenic conditions such as acne, rosacea, and atopic dermatitis. In addition, the importance of maintaining a balanced bacterial population to ensure skin wellness and preventing the proliferation of pathogenic bacterial species thanks to the available treatments present on the market is taken into account.

For this purpose, studies conducted in order to highlight the bacterial composition in the presence of the pathologies mentioned earlier are reported.

## 2. Literature Search Methodology

Articles were searched in databases such as Science Direct, PubMed, Medline, and Google Scholar with keywords including skin microbiota, skin disease, atopic dermatitis, acne vulgaris, rosacea, and all other keywords associated with the topic. The most relevant articles published from 2000 to 2022 were read to write this review. The studies selected include patients of both sexes, regardless of age.

The first step included the examination of titles and abstracts; then, articles including analyses of skin microbiota and skin disease were selected. The next step was the comprehension and the screening of full texts according to the inclusion criteria: microbiota analysis in atopic dermatitis, acne vulgaris and rosacea patients, and studies focusing on the biophysical skin parameters. Finally, only the most relevant scientific papers were considered.

## 3. Atopic Dermatitis

**Figure 2:**
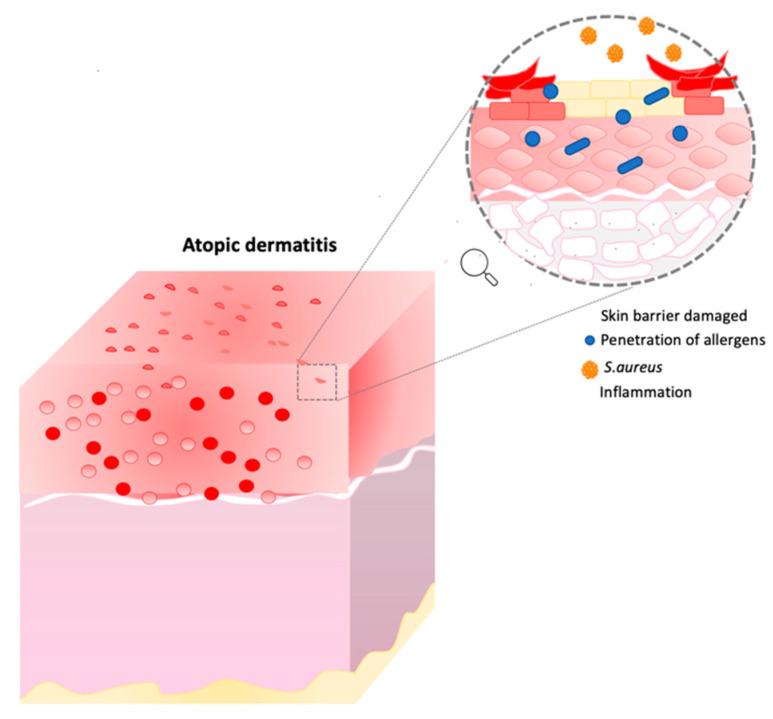
Atopic dermatitis representation. This image describes the consequence of skin barrier damage. Image built on Power Point software.

Atopic dermatitis is a chronic inflammatory skin disease in which the major symptoms are xerosis, dry itchy skin and eczema. In this condition, the skin barrier is compromised, and individuals are more exposed to secondary infections, penetration of allergens, and transepidermal water loss (TEWL) [17,18,19]. Moreover, other characteristics such as inflammation, immune dysregulation and filaggrin mutation can affect AD patients (Figure 2) [19,20,21]. 

### 3.1. Mutation of Filaggrin

Among the causes we can find in AD patients, there could be a mutation in the gene (FLG) encoding for the protein filaggrin, localized on the short arm of chromosome 1q21 [22]. Filaggrin is a structural, S100 calcium-binding epidermal stratum corneum (SC) protein. It is involved in the normal SC function in terms of the hydration and maintenance of the skin barrier. It is also responsible for the binding of the keratin filament to produce micro-fibrils. During epidermal differentiation, its insoluble precursor, profilaggrin, is dephosphorylated and becomes more soluble [23]. Then, different proteases cleave the molecule into monomers, forming the final structure [6,24,25]. The role of FLG is to generate natural moisturizing factors (NMFs) and to provide a scaffold for the extracellular lipid matrix. If filaggrin is not synthetized correctly or if there is a mutation, then the differentiation of keratinocytes will be dysregulated, and the barrier will be compromised. Not only keratinocytes are involved, but lipid alteration, a reduction in moisturizing agents and barrier disruption are other characteristics present in AD. The consequence of these factors is that the skin is exposed to different infections and pathogens. Moreover, if the SC barrier is not structurally uniform, then keratinocytes are not able to create a compact layer, and for this reason, the epidermis will have an increase in transepidermal water loss (TEWL), while skin hydration and the level of NMFs will be lower [26]. 

It is important to point out that FLG mutations are neither necessary nor sufficient to cause atopic dermatitis; in fact, 60% of patients do not have this mutation [27]. 

Not only genetic mutations, but other effects, such as the inflammation process and microbiome imbalance, can also contribute to AD development. 

### 3.2. Inflammation Process

AD is a combination of two pathologies: a skin barrier deficit, such as a filaggrin mutation, and an immune dysregulation. Different hypotheses have been reported; the first suggests that immunological aberrations are the primary phase leading to the development of the disease, with the skin barrier being affected. The second one states that a compromised epidermal barrier primarily leads to the onset of topical eczema and secondarily to immune dysregulation [28]. 

In particular, AD follows a two-step process: from an acute to a chronic state. In the initial, acute phase, T-helper 2 and T-helper 22 cell responses are increased in the skin, with some involvement of T-helper 17 cells. The mediators produced can influence skin inflammation, and they contribute to the impairment of the skin barrier. Moreover, they activate different cell types, such as keratinocytes, which increase skin inflammation through the release of proinflammatory cytokines. The disease continues its progression in a chronic manner, in which type 1 immunity prevails with Th1 pathways and a still important contribution from T-helper 2 cells [29,30]. 

### 3.3. Microbiome in AD

In addition to genetic evidence, the microbiota also plays a key role in the development of skin diseases. In fact, the perturbation of resident flora can determine the colonization of some pathogenic species. A diverse composition of the microbiota can also alter the epidermal barrier. 

The bacterium present in the highest percentage in atopic skin is *S. aureus*. It is able to produce toxins, enzymes and antigens capable of bypassing the immune system and the skin barrier. Its enterotoxins can induce the expansion of T- and B-cells, leading to the production of inflammatory cytokines., e.g., toxins and leucocidins. They initiate the process of cytokine production, hemolysis and leukocyte death. β-toxin, on the other hand, causes cell death, inflammation and destruction of the skin barrier. We can say that these substances help *S. aureus* to grow and survive by targeting the host’s immune response and the integrity of the skin barrier [31,32,33].

A study conducted by Amy S. Paller et al. confirmed that in AD, an over-colonization of *S. aureus* (Gram-positive aerobe bacterium) occurs [34]. Thanks to epidemiological, metagenomic and functional studies, it has been shown that the link between this bacterium and AD is sophisticated and depends on host and pathogen factors. While some host factors (physical, antimicrobial) aid in the maintenance of healthy skin, this bacterium can adhere and invade the epidermis, contributing to and promoting the development of an inflammatory state [35].

In an atopic patient, in fact, the percentage of this bacterium is found to be higher, with an increase from 30 to 100% depending on the type of patient, the size of the sample site taken, and the method used for microbial analysis. In the literature, different sampling methods have been observed to analyze the bacterial composition. However, heterogeneous methods have led to different results. 

Other results have suggested that when analyzing the skin microbiota with a deep shotgun metagenomic sequencing, the microbial diversity is lower during an AD flare. In particular, in inflamed atopic skin, the genera *Streptococcus, Corynebacterium*, *Cutibacterium* and the phylum *Proteobacteria* decreased with respect to the genus *Staphylococcus* [35]. 

Instead, if we consider bacterial-culture-based methods, a meta-analysis including 95 studies demonstrated a different distribution of this bacterium on the same patient in injured areas (70%) than in the uninjured areas (39%) [36]. 

In fact, there is still no real cure that targets the different bacterial composition in AD, and for this reason, possible future therapies could be those in which the precise target is the skin microbiota, with the aim of restoring the commensal species to bring back a healthy and balanced skin environment [37]. 

Other studies were performed to investigate the microbiota in AD. For example, Kwon et al. [38] evaluated differences in the skin surface microbiome in 18 patients (aged 5–40 years old), prescribing TCS (methylprednisolone cream) and oral histamine. The microbiome was compared between lesioned and non-lesioned skin at different time intervals. The results showed that the proportion of the genus *Staphylococcus*, which was >80% in baseline lesioned skin, decreased drastically after treatment (week six) and increased slightly after the discontinuation of treatment (week nine). Moreover, the proportion was much higher in lesioned than in non-lesioned skin, even after treatment. In baseline lesioned skin, *S. aureus* comprised 72.5% of the total species, while *S. epidermidis* and *S. caprae* were the second and third most common species, respectively. In non-lesioned skin, *Cutibacterium acnes* was the most common species, followed by *S. aureus* and *S. epidermidis*. The proportion of *S. aureus* on lesioned and non-lesioned skin was significantly different (*p* = 0.0014). As a result, we can state that this bacterium was more present in lesioned skin at all time points, including week six. The proportion of *S. epidermidis*, on the other hand, in baseline lesioned and non-lesioned skin was 6.3% and 7.1%, respectively. 

This study highlighted how the microbiota changes under injury conditions. In fact, it confirmed that in AD with wounds, *S. aureus* is the species that predominates with respect to non-lesioned skin, where the composition is different and where *Cutibacterium acnes* prevails. Moreover, Kwon et al. demonstrated that topical cream, such as TCS, in combination with oral histamine can decrease the colonization of *S. aureus.*

Previously, we stated that different sampling methods can lead to different results. The most common method used is swabs (as also reported in this study), which allow an analysis of the surface microbiota. Other methods (for example, strips as a non-invasive method, and skin biopsies as a more invasive method) can be used to study the microbial composition in the deeper layers of the epidermis.

In the work of Martin et al. [39], 23 children (six months old with a familiar predisposition) were enrolled. This study aimed to investigate skin parameters and microbiota composition changes with (*n* = 11) or without (*n* = 12) the use of an emollient. Parameters were evaluated only after six months. For the skin results, pH and TEWL were measured. In both cases, they were lower in the emollient group than the control one, while the skin water capacitance was higher in the emollient group [38]. The lower value of TEWL should be considered as a positive aspect, as this correlates with the restoration of the skin barrier and the firmness of corneocytes. 

The main shortcoming of these studies is related to the duration of treatment. In fact, since the use of the product lasted for only six months, it is not possible to predict the long-term effects [38]. For example, we cannot affirm if, with the application of the product, the barrier function will be restored completely. For microbiota analyses, skin samples were obtained by using a flocked swab. Bacterial DNA was amplified and later sequenced, targeting the 16S rRNA gene, in particular regions V1-V3, which allowed the bacterial discriminations. The *S*. *mitis* group and *S*. *salivarius* were shown to be present in different compositions between samples from the emollient and control groups, while *Streptococcus* predominated in both the emollient and the control groups. 

*S*. *salivarius* was significantly higher in the emollient group than in the controls at all sampling sites (cheek, *p* = 0.02; dorsal forearm, *p* = 0.02; volar forearm, *p* = 0.02); instead, *S. mitis* was less shown in the emollient group than in the controls, without any particular significance [38,39].

The next step was the analysis of *S*. *salivarius* in the healthy neonates and in patients with AD in the control group. The proportion of this bacterium in the cheeks appeared lower in AD infants as compared to healthy ones. The decrease in the *S*. *salivarius* proportion associated with AD was not statistically significant, presumably due to the low number of infants with AD (*n* = 3). 

Although the limitation of this study has already been mentioned, the hypothesis is that the long-term use of the product with emollient effects, leading to a decrease in pH and an increase in *S. salivarius*, may help children with atopic dermatitis to avoid the onset of more serious symptoms. 

With these studies, it can be considered how different analysis methods and microbiota sampling can lead to different results. In fact, if in the first study we focus on *Staphylococcus aureus* as the main exponent present in a higher percentage of AD patients, then in the last study, *Staphylococcus salivarius* and *mitis* are emphasized as the major species. As a result, it can be stated that it is necessary to find a standardized sampling method to analyze the bacterial composition, and thus the skin microbiota at a deeper level, that is, not focusing on species only but also the subspecies and phylotype levels. In this way, we can better understand what the real players are that are present on an atypical skin sample, identifying the genetic characteristics.

Treatments for this therapy are complex, and they vary depending on the patient’s condition, age, compliance, and cost. Treatments are also divided into topical and systemic. Firstly, the use of emollients and moisturizers is intended to provide hydration, repair the skin barrier and reduce itching, redness and flaking [40,41,42]. 

These products usually contain a high percentage of oils. Another recommendation is to take baths once a day for 5 to 10 min in lukewarm water. In more severe cases, dermatologists suggest adding bleach (0.005%) twice a week to counteract the over-colonization of *S. aureus*, as it has antiseptic properties. If we consider medication, the first line of treatment is corticosteroids, which have an anti-inflammatory effect on immune cells such as T-cells and macrophages. Different types of corticosteroids can be used depending on the stage of the disease: in more severe cases, class I corticosteroids are recommended, while in milder cases, patients can take class VII corticosteroids. The latter are often applied in children or in more sensitive areas of the body, where the skin is thinner. These drugs are suggested as they are able to contrast the growth of *S. aureus* by inhibiting the production of the peptides it produces. Doctors usually recommend brief use because they can lead to side effects such as redness, striae, and atrophy [43,44]. 

The routes of administration can be different. Indeed, they can be applied directly to atopic skin, or delivered through wet wraps, allowing the active ingredient to penetrate more deeply, at the same time providing protection to compromised skin and lowering transepidermal water loss. In the most extreme cases, topical application is not sufficient. Consequently, the systemic administration of immunomodulators such as cyclosporine and azathioprine is used. The last approach, which could be considered as an adjuvant, is phototherapy. For this purpose, UVB irradiation (NB-UVB/UVB 311 nm) and medium-dose ultraviolet radiation (UVA1) are used [45,46,47]. 

## 4. Acne Vulgaris

Acne vulgaris is a chronic phlogistic disease that mostly affects adolescents (between 35% and 90%). It is represented by skin changes such as seborrhea, non-inflammatory lesions (comedones) and inflammatory lesions (papules and pustules) [48]. It mainly occurs in the pilosebaceous units and can develop in different forms based on the number of abnormalities, nodules, cysts, and abscesses [46]. The main features of acne correlate with an overproduction of sebum, an abnormal process of keratinization, *Cutibacterium acnes* colonization in oily body sites (face, neck, chest and back), and an inflammatory process. As in AD, there is no clear information regarding the interactions and order of events that occur for the development of this disease [47].

There is increasing evidence on the role of diet in acne. High glycemic index diets, dairy consumption, and whey protein consumption have been called into question. Dietary modifications and natural treatments are likely to play an increasingly important role in acne treatment as more evidence accumulates [48,49,50,51,52]. 

Different pathological processes correlated with different cells contribute to the development of acne. In sebocytes, for example, hyperseborrhea and diseborrhea occur. These phenomena result in an alteration in sebum composition. Moreover, an over-colonization of *Cutibacterium acnes* with the formation of biofilms aggravates the condition. Indeed, the biofilm makes the bacterium more resistant to treatment. 

Focusing on sebocytes, androgens stimulate their differentiation and proliferation. The mechanism allows the hormones to bind to their receptors, androgen receptors (ARs), that cause the phosphorylation of mTOR. mTOR, and then the activated mTORC1, stimulate lipogenesis with the increase in lipids in cells. Other factors, such as CRH, α-MSH and substance P, are involved in sebocyte activity. However, the mechanism of α-MSH with its lipogenetic effect remains unclear. Substance P, instead, is a stress-associated neuropeptide, and it increases the activity of IL-1, IL-6 and TNF-α. 

Androgens are also linked with c-MYC expression via the Wnt/beta-catenin pathway for sebocyte differentiation [53,54]. 

Other cells that are involved are keratinocytes. When IL-1 binds with its receptor (IL- 1R), this causes hyperkeratinization. Another factor that aggravates this process is the altered sebum composition. 

Moreover, keratinocytes are also involved in the inflammatory response with the presence of *C. acnes*. Indeed, they activate TLR 2 and TLR 4 by activating the NF-kB and MAPK pathways. Then, the cells release the inflammatory cytokines and immune cells, such as macrophages, and stimulate the production of TNF-α and β-defensin. 

In addition, when the bacterium is perceived by CD36, it produces reactive oxygen species (ROS), increasing the inflammatory process. In addition, neutrophils are involved in the production of hydrogen peroxide [55,56]. 

Various treatments are available for acne vulgaris. Usually, combination therapy is used rather than systemic therapy, in which other substances such as retinoids or benzoyl peroxide are prescribed along with antibiotics. 

Topical treatments have a keratolytic function, capable of disintegrating the desmosomes and hemidesmosomes to correct abnormal keratinization. Different classes with different concentrations, depending on the severity of the disease and the duration of treatment, are available [56]. 

The gold standard for the topical treatment of acne is benzoyl peroxide, introduced in 1930. Its function concerns oxidation and the formation of free radicals by reducing *C. acnes* colonization. It is used for the mild and moderate stages of the disease and has antimicrobial, anti-inflammatory and anti-comedogenic effects. Benzoyl peroxide, once applied, is absorbed into the epidermis, and it is converted into benzoic acid. Its hydrophobic characteristic allows it to accumulate in the sebaceous units, thus fulfilling its function. It is available in different concentrations between 2.5 and 10% [56]. 

Patients using this drug often complain of dryness, burning and redness. It is usually combined with antibiotics such as erythromycin and clindamycin. 

Another class of topical substances used are retinoids, which also have similar effects to benzoyl peroxide. They exert their function of regulating the cohesion and adhesion of keratinocytes by breaking up the corneal plug. The mechanisms of action are multiple and include normal epidermal proliferation and differentiation, the inhibition of neutrophil chemotaxis and secretion of proinflammatory cytokines, and downregulating TLRs [56,57]. 

This approach is optimal in patients with both inflammation and comedones. In addition, retinoids, by decreasing the adhesion of the corneocyte barrier, facilitate the entry and permeability of antimicrobial agents, allowing the entry of antibiotics.

Examples of retinoids used are azelaic acid, salicylic acid, tazarotene and adapalene. Azelaic acid, for example, modifies epidermal keratinization, acts against aerobic and anaerobic bacteria, has anti-inflammatory activities, and inhibits ROS formation.

Instead, salicylic acid has a greater effect on the stratum corneum (SC), destroying the cohesion and desquamation of keratinocytes. The use of 0.5–2% salicylic acid reduces acne inflammation and closes and opens comedones in 12 weeks. There are new chemical peels using 30% SA delivered with polyethylene glycol that show efficacy and safety with a drastic reduction in comedones and papules [56,57]. 

Tazarotene is hydrolyzed into tazarotenic acid by keratinocytes. It has anti-comedolytic, anti-comedogenic and anti-inflammatory functions. Using gel with 0.05–0.1% tazarotene reduces non-inflammatory acne lesions in 12 weeks. 

Comparing the cream with 0.1% tazarotene with a cream containing 0.1% adapalene, it has been shown that tazarotene is more effective in reducing comedones, showing no side effects over 12 weeks.

Topical antibiotics include clindamycin, erythromycin and tetracyclines. Among them, erythromycin has been found to be the topical antibiotic with the greatest efficacy. It binds to the 50 S ribosomal unit, and it prevents the translocation of peptidyl-tRNA from the A-site to the P-site that is necessary for protein synthesis in bacteria. It is active against *C. acnes* and it reduces the colonies on the skin surface and within follicles. It has been considered a very effective topical antibiotic in acne therapy, but it was recently discovered that *C. acnes* is up to 60% resistant to erythromycin, making it less suitable. This has sparked interest in the future development of other topical antibiotics. The problem of *C. acnes* resistance towards clindamycin and erythromycin is due to mutations at the 16S and 23S rRNA level, which confers cross-resistance to antibiotics such as macrolides, lincosamides and B-type streptogramins [58]. 

Tetracyclines with broad-spectrum activity and bacteriostatic action, on the other hand, bind to the 30S subunit. 

For systematic delivery, oral antibiotics are available. In particular, they are indicated for moderate and severe acne and for patients in whom topical treatments have failed. The success of antibiotic treatment is based on the ability of the agent to reach the lipid environment of the pilosebaceous follicles in the dermis, and so to reach *C. acnes*. Tetracyclines are widely used because they are effective and inexpensive. Doxycycline and minocycline are preferred because they cause less gastrointestinal irritation and are more liposoluble, so they penetrate the hair follicle more effectively. 

To reduce resistance and improve efficacy, oral antibiotics should be combined with topical benzoyl peroxide or retinoids. Furthermore, the duration of treatment should not exceed 12 weeks whenever possible [59]. 

One particular drug for emergencies is isotretinoin, which is used for the most severe cases when no other therapy is effective. Its action is explicated on the sebaceous glands, decreasing their size and secretion. It also has anti-comedogenic activity and reduces the proliferation of *C. acnes.* This treatment lasts from 16 to 24 weeks, but regular patient check-up is necessary due to the side effects that may occur. In fact, isotretinoin is a teratogen and should not be taken during pregnancy or while breastfeeding. Other undesirable effects include bone weakening, increased sensitivity to the sun, and an influence on blood sugar and night vision [60,61]. 

### 4.1. Cutibacterium Acnes 

The key role is played by *Cutibacterium acnes* [62,63], a Gram-positive, lipophilic, and anaerobic bacillus that mainly resides in the oily sites of human skin such as pilosebaceous glands. Several mechanisms of acne pathogenesis including this bacterium have been discussed [64]: an increase in sebum production, promotion of comedone formation and induction of inflammation.

Firstly, *C. acnes* is implicated in lipid metabolism, in particular, in the hydrolysis of the sebum’s triglycerides, and in the production of free fatty acids that are responsible for the acidification of the skin [65,66,67]. The quality and quantity of lipids on the skin are related to the colony-forming units (CFUs) in the pilosebaceous unit. *C. acnes* uses the sebum as a substrate to support its expansion and enhances the activity of diacylglycerol acyltransferase and sebum secretion [68]. With its over-colonization, the composition of lipids changes: the decrease in linoleic acid, the increase in sebum, and the increase in monounsaturated fatty acid (MUFA) expand the comedogenesis process [69]. 

Moreover, C. *acnes* secretes different types of enzymes, such as lipases, metalloproteases, and catalytic factors such as porphyrins. These molecules react with oxygen, generating toxic species, damaging keratinocytes and causing the oxidation of squalene. 

This bacterium is also involved in the innate immune system, activating TRLs and then pathogen-associated molecular patterns (PAMPs). PAMPs in turn are recognized by DAMPS (damage-associated molecular patterns), creating inflammasomes and activating caspase 1, IL-1beta and IL-18, which are responsible for papules on the individual. 

**Figure 3:**
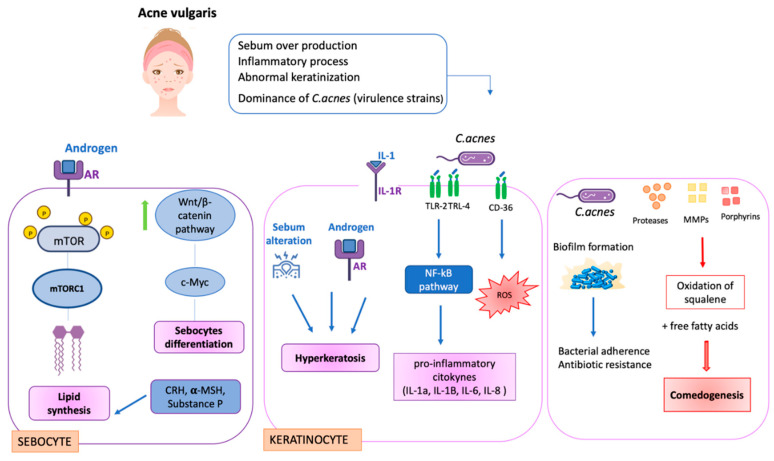
Illustration of *Cutibacterium acnes’* mechanism of action. Made with Power Point software.

Different studies in the literature have demonstrated that *C. acnes* plays an important role in the innate immune response: it binds to TLR 2 and TLR 4 on the surfaces of keratinocytes and induces monocytes and other cells to secrete proinflammatory cytokines and proteins. Moreover, it can interact with the classical and alternative complement pathways, resulting in the circulatory permeability and recruitment of leukocytes being increased [67,68,69,70,71,72] (Figure 3). 

In acne pathogenesis, insulin-like growth factor 1 and the filaggrin pathway are over-expressed, provoking an increase in different integrins (α-3, α-6, and vβ-6) and influencing keratinocyte proliferation and differentiation. 

Therefore, free fatty acids, the oxidation of squalene, and the expression of integrins and cytokines are the main factors that promote the progression of acne. Follicular hyperkeratinization in the sebaceous gland and follicular infundibulum can be considered some of the essential requisites for the development of acne lesions [73,74]. 

However, *Cutibacterium acnes* is not only a pathogen responsible for acne vulgaris, but it is also a commensal bacterium. The latest research has allowed the differentiation of different strains involved in the pathology. Considering the genomic analysis of the recA gene, *C. acnes* can be divided into four phylotypes: IA, IB, II and III [75,76]. A more in-depth approach (multi-locus sequence typing MLST), which is instead based on nine housekeeping genes, has further sorted phylotype 1 into IA1, IA2, IB and IC. Phylotype IA1 was found in acne patients, while phylotypes IA2, IB, II and III were mainly found in the healthy group [77,78,79,80]. This evidence demonstrates that only certain strains correlate with the development of acne vulgaris, while the others are fundamental to maintaining healthy skin.

### 4.2. AV Microbiome Studies

In this section, different studies are reported to investigate the different aspects of acne vulgaris, such as how physical parameters and the microbiota change. 

Firstly, a work by Yamamoto et al. [81] focused on the analysis of several parameters, comparing patients with and without acne. They observed that in acne patients there was an increase in TEWL and a lowering of conductance in the stratum corneum. Moreover, sebum production was enhanced, compromising hydration levels. They also evidenced that the composition of lipids was altered in the intercellular membrane; the levels of sphingolipids (ceramides and free sphingosine) were particularly reduced. They concluded that the increased TEWL and reduced hydration of SC are some of the events directly connected with the disease severity. 

Li CX et al. investigated 67 patients with different degrees of acne, with the aim to study the possible difference in bacteria composition related to the various stages of the disease. The method chosen was 16S rRNA gene sequencing [82]. As they reported, the main bacterial phyla found in both groups (patients and healthy controls) were *Actinobacteria, Firmicutes, Proteobacteria* and *Bacteroidetes*, while if we consider the genera classification, *Cutibacterium, Staphylococcus, Streptococcus, Corynebacterium* and *Lactobacillus* were present in a homogeneous ratio without discrimination. 

The positive aspect of this study was the discovery of a diversity of bacterial composition correlated with the different stages of the severity of acne. In fact, if the main phyla and genera were present without distinction in both groups, then a different microbial composition was observed among acne patients. For this reason, the study divided patients with grade 1, 2, and 3 acne from patients with more severe acne, classified as level 4. The most prevalent genera present in patients with a more severe stage of the disease were *Enhydrobacter, Bacteroides, Faecalibacterium, Klebsiella, Oscillospira, Ruminococcus* and *Escherichia*, while *Faecalibacterium, Klebsiella, Odoribacter* and *Bacteroides* represented the genera whose percentage was much higher. In contrast, when considering patients with acne 1–3, there was no significant difference at the gender level [82].

The limitation of this work regards the bacterial analysis, because it would have been interesting to analyze the bacteria species and subspecies to investigate the differences between subjects related, for example, to different types of *C. acnes.*

As in previous studies on AD, the studies reported here do not focus on a more in-depth analysis, but in this case dwell only on the bacterial genus levels. This certainly does not facilitate a broad-spectrum understanding regarding the disease–microbiota alteration correlation, but evidences a superficial analysis.

In addition to the analysis of the skin parameters and microbiota, studies have also been carried out using active ingredients.

For example, in the study of Lubtikulthum P. et al., experimental work was performed using two different active ingredients. For this purpose, a randomized controlled trial consisting of 77 subjects was conducted. The participants were asked to apply a natural herbal active (HPE) or the 2.5% benzoyl peroxide (BP), used as the gold standard in acne therapy, for 84 days [83]. After the application period, several parameters were evaluated: the reduction in comedones and inflammatory lesions. 

The results obtained did not show significant differences (there was a 39.4% reduction in comedones with benzoyl peroxide and a 34.51% reduction with the application of the natural active ingredient, as well as a 40.9% reduction in inflammatory lesions with BP and a 40.54% reduction with HBP). 

The average number of porphyrins was also evaluated. As mentioned before, these proteins appear to be important catalytic factors produced by *C. acnes* and are involved in tissue damage and inflammatory stages. Even in this case, in both treatments there was a significant decrease from 1511.17 ± 1126.23 to 1204.45 ± 765.73 (*p*-value = 0.003) in the HBE group and from 1815.73 ± 1313.87 to 1397.43 ± 916.20 (*p*-value < 0.001) in the BP group.

No systemic adverse effects occurred. Already, these data suggest that the natural extract can be used as a treatment for mild and moderate acne for those patients who cannot tolerate the benzyl peroxide drug due to skin reactions such as dryness and desquamation. An important aspect is that both groups reported a better quality of life after the application of the products, and this result is an indicator of the benefit of the drug. HBE can be used as an alternative medicine instead of BP [83]. These studies demonstrate that different approaches can be used to develop new treatments for acne vulgaris. For example, using novel antimicrobial peptides or natural actives in order to limit the use of antibiotics can alter the microbe balance. 

## 5. Rosacea

The last pathology considered in this review is rosacea, a disease that mainly affects the areas of the face. Like acne vulgaris and atopic dermatitis, it has a high incidence worldwide: it affects between 0.9% and 10% of the population, mainly women over 30 years old. Since the causes are not clear, the therapies available on the market are not specific, and in fact dermatologists tend to simply prescribe cortisone or, in the worst-case scenario, a veterinary antiparasitic, ivermectin. It is thought that the etiological factors that determine the onset of this condition are neurovascular and immunological mechanisms [83,84]. 

The National Rosacea Society (NRS) expert committee attributes redness, persistent erythema, papules, pustules, and telangiectasias as unambiguous signs related to this disease. Based on the clinical characteristics, rosacea can affect different sites of the face: it can occur on the forehead, cheeks, nose and chin (subtype 1: erythematotelangiectatic rosacea), manifest only on the cheeks (subtype 2: papulopustular rosacea), affect the nose (subtype 3: phymatous rosacea), or begin around the eyes (subtype 4: ocular rosacea). These conditions can develop individually or together, resulting in a more serious and certainly annoying stage of the disease for the patient [84].

The origin of rosacea development is not clear, but several factors and elements, ranging from microbial, genetic and immunological causes, are involved. The dysregulation of innate and adaptative immunity, chronic inflammation and aberrant neurovascular signaling are principles that can affect patients. Studies suggest that neuroinflammatory responses and the immune system contribute to the outbreak of rosacea [85].

Under healthy conditions in the innate immune system, a key role is played by TLR receptors, which are expressed on the surface of skin cells such as keratinocytes and macrophages. The activation of these receptors (by chemical and pathogenic stimuli) results in the production of inflammatory molecules (cytokines and chemokines) and antimicrobial peptides. In rosacea patients, TLR 2 is overexpressed, leading to increased skin sensitivity to external factors and to the production of IL-8, IL-1β and TNF-α. The presence of other molecules such as caspase 1 worsens the inflammatory response, activating other factors such as metalloproteases. The adaptive immune system, on the other hand, is not fully known. It has been observed that a role could be played by α- and β-defensins, which induce the chemotaxis of T-lymphocytes and stimulate antibody production by B-lymphocytes. In skin biopsies of patients, the involvement of B-lymphocytes and plasma cells has been observed, resulting in increased levels of CD20 (B-cell marker).

In addition to abnormal immune regulation, neuronal and vascular control is also implicated in the process. The problem of the nervous system leads patients to be more exposed and susceptible to changes in temperature, stress and ultraviolet radiation. In these subjects, the level of vascular endothelial growth factor (VEGF) and non-selective calcium permeable ion channels (TRP) are highly expressed; in particular, TRPV-1 has proinflammatory properties, and it is co-involved in vasoregulation, leading to acute and chronic pain; TRPV-2 is implicated in vasodilation and TRPV-3 in inflammation. TRPV receptors and the neuropeptides produced are linked to the immune mechanism through neurogenic inflammation. The neuropeptides PACAP, SP, and VIP regulate the main immune responses such as antibody production, lymphocyte activity and cytokine secretion. They also participate in vascular permeability, extravasation, and in the vasodilation process [86]. 

Focusing our attention on the microbiota, in the literature, most of the evidence is related to parasites, such as *Demodex* [86,87]. In fact, several studies have been performed to attenuate the manifestations of rosacea and to compare the behavior of the skin microbiota with different treatments.

### RS Microbiome Studies 

Ezgi Akta ş Karabay et al. [88] investigated whether the presence of *Demodex mites* is associated with a pathogenic role when it is found in high density. This work focused its attention on both rosacea patients and acne vulgaris and seborrheic dermatitis patients. A controlled study was performed with the recruitment of 42 patients with acne, 43 patients with rosacea and 41 patients with seborrheic dermatitis. These were compared with 77 healthy subjects. The method of analysis was a skin biopsy, and samples were taken from the cheeks and upper forehead. The results showed that in terms of gender and age, there were no significant differences. As for the patients, 52.0% showed a *Demodex* infestation compared with 2.6% for the controls. *Demodex* infestation rates were increased in patients compared with the controls (*p* = 0.001). Subsequently, comparing only the patients divided by disease category, it was noted that the presence of *Demodex* was much more clearly present in the rosacea group (79.1%) than in the AV (27.9%) and SD (48.8%) groups.

Then, the authors wanted to investigate the role of two *Demodex* species: *D. folliculorum* and *D. brevis*. These are usually found on healthy skin; in particular, the first resides in the hair follicle, while the second is in the sebaceous glands [89,90]. The activity of these microbes is correlated, as in the case of *C. acnes*, with the metabolism of lipids and, in particular, the sebum, which is used as nourishment to enhance their growth [91].

In the work of Draelos ZD et al. [92], the effect of a topical antibiotic (3% monocycline) on the skin barrier was evaluated. For this purpose, 31 subjects of both sexes, with variable complexion and moderate rosacea, were enrolled. The two biophysical parameters considered were transepidermal water loss (left cheek) and hydration (right cheek). The measurements were taken on day 1, after 15 days and subsequently after 1 month. The study required the application of the product every evening. The results suggested that the product is able to restore the skin barrier as TEWL decreased over the duration of the study, with an 11% reduction on day 1, followed by an 18% reduction in week two (*p* = 0.001) and a 28% decrease by week four (*p* < 0.001). Promising results were also obtained for hydration: the gel demonstrated a 23% increase (*p* = 0.003) on day 1, a 22% increase (*p* = 0.003) in week two and a 20% increase (*p* = 0.001) in week four. These findings indicate that the study medication produced no irritation in subjects with rosacea-induced sensitive skin and functioned as a moisturizer to increase skin hydration.

A quality topical formulation will decrease TEWL and increase skin hydration immediately with continuous improvement over time, as demonstrated by this research on an investigational topical minocycline anhydrous gel of 3%. After a single application of the newly investigated minocycline gel on day 1, TEWL dropped by 11% and corneometry increased by 23%. By the end of four weeks of use, TEWL dropped by 28% with a corneometry increase of 20%. This shows that once-daily use of the investigational topical minocycline anhydrous gel of 3% continued healing the skin barrier with maintenance of skin moisturization. Thus, the formulation not only significantly decreased the number of inflammatory lesions and significantly improved the Investigatory Global Assessment (IGA) score in patients with a papulopustular rosacea disease burden, but also improved the skin barrier, facilitating optimal healing [93]. 

Ebneyamin E. et al. instead evaluated the efficacy of 2.5% permethrin in combination with tea tree oil (TTO) to observe variability in parasitic density, particularly for *Demodex*. The study was conducted in a double-blind manner. Thirty-five patients were enrolled, and the design involved the application of the active ingredient on one side of the face and the placebo on the other area, twice a day for a period of 84 days. The results showed that the percentage of mites decreased significantly compared to the placebo (*p*-value = 0.001) [94]. On day 1, the amounts of *Demodex* were 1407.09 (placebo zone) and 1345.99 (active zone). After 12 weeks, the parasite number lowered to 650.94 for the placebo and 528.77 for the active-treated zone.

So, we can conclude that the drug was more effective than a placebo in reducing mite density after five weeks. In addition to parasite density, the authors evaluated the main manifestations in both groups, demonstrating that there were no statistically significant differences (*p* > 0.05). In fact, signs such as erythema, plaques and edema were present in both. Subsequently, an analysis of these characteristics was performed for each group involved. It was observed that papules and pustules improved, unlike plaques and edema, which did not show any differences. After 12 weeks of treatment, symptoms such as burning and dryness decreased compared to the placebo, which also resulted in a better quality of life for the patient. [88]. To quantify the density of *Demodex,* a skin biopsy was used on patients after washing their faces with warm water and no soap. Once the skin sample was taken, it was studied with a magnifying microscope, and the number of mites determined the density of *Demodex*.

Thee results show that permethrin 2.5% with tea tree oil can easily reduce the *Demodex* population with respect to the placebo control, so it could be a possibility to restore the bacteria composition, in terms of *Demodex* density, and to improve the balance of patients affected with rosacea or other diseases in which this mite could be involved. 

## 6. Conclusions

This review aims to demonstrate that the scientific connection between the skin microbes and the onset of skin diseases is still unclear. The studies that were taken into consideration to investigate the composition ranged from the genus to the species level. However, future studies could investigate the correlation of the microbiota and skin dysbiosis at the subspecies and strain levels to observe, as in the case of acne vulgaris, whether particular strains of a given bacterial species are more involved.

Moreover, it is necessary to set up a universal standardized protocol for in vivo sampling of the microbiota. In fact, although the most-used method is swabs, these only allow for superficial microbial analysis, while skin biopsies, although working in the deeper layers of the skin, are invasive. For this reason, further studies are needed to define the microbiota sampling method, even trying to correlate the presence of bacteria with biophysical parameters such as hydration, TEWL, sebum, erythema and porphyrins to gain a global and deeper knowledge of the disease. In fact, with regard to the diseases addressed in this review, few data are available regarding the bacterial population. In fact, for atopic dermatitis only, an over-colonization of *S. aureus* is referred to, while for rosacea there is still no precise scientific evidence.

Moreover, since it has been observed that *C. acnes* acts as a protagonist in acne, the next steps could be to investigate not only the biofilm that this bacterium forms in sebaceous glands, but also the active ingredients that penetrate inside, which have an antibiofilm effect, to eradicate the over-colonization of the pathogenic specie. 

In the studies reported in this review, reference is also made to other types of products that can be used in place of retinoids or antibiotics, such as a natural extract (HBE) in the case of acne vulgaris, or simply an emollient to reduce the adverse effects of atypical skin. In this way, we can think to reduce the use of retinoids, which, despite being the gold standard for the treatment of acne vulgaris, can lead to side effects such as burning, dryness, and peeling in certain patients.

Retinoids are the first approach used worldwide for the treatment of acne. All the lines and recommendations of dermatologists and skin experts support the use of these substances, especially for acne vulgaris. In fact, the problem arises not because of the use of them, but the method of using them. In fact, it would be possible to use retinoids at a lower concentration only for patients who tend not to have irritation and burning, while therapy can be prescribed to those patients who tolerate this type of substance very well, which brings more benefits than drawbacks. In certain situations, antibiotics could be substituted with other substances, possibly following specific guidelines, without using them as the first line in the treatment of these diseases, so as to avoid harming the microbial population present on the skin. Consequently, we can consider an approach aimed at first studying a thorough level of bacterial species and especially bacterial subspecies involved in the pathogenesis of the disease, and then studying and finding alternative treatments that can improve the appearance of pathological skin in those patients who are sensitive to current therapies, finally attaining specialized therapy for each patient. 

Special attention must be paid to rosacea, in which the causes are still unknown, and for treatment or to alleviate the symptoms, a drug for veterinary use is often prescribed that can result in major side effects.

## Data Availability

Not applicable.
